# From Phytochemistry to Metabolic Regulation: The Insulin-Promoting Effects of Loureirin B Analogous to an Agonist of GLP-1 Receptor

**DOI:** 10.3390/ijms262311548

**Published:** 2025-11-28

**Authors:** Haowen Fang, Xiaodong Sun, Yanting Ding, Siyuan Gu, Bing Niu, Qin Chen

**Affiliations:** 1 School of Environmental and Chemical Engineering, Shanghai University, Shanghai 200444, China; fanghaowen@shu.edu.cn (H.F.);; 2 School of Life Sciences, Shanghai University, Shanghai 200444, China; zhukaoguan@shu.edu.cn; 3 Shanghai Engineering Research Center of Organ Repair, Joint International Research Laboratory of Biomaterials and Biotechnology in Organ Repair (Ministry of Education), School of Medicine, Shanghai University, Shanghai 200444, China; sunxiaodong_cpu@163.com

**Keywords:** diabetes, drug design, GLP-1R, pharmacokinetics, drug discovery

## Abstract

This study aimed to develop a potent, safe, and cost-effective small-molecule hypoglycemic agent derived from Loureirin B, with preliminary evaluation of its efficacy and mechanistic underpinnings. Thirty structural analogs of Loureirin B were synthesized. Molecular docking identified LB-A as the lead compound targeting GLP-1R. Its hypoglycemic activity was initially assessed in a murine model. Molecular dynamics simulations, surface plasmon resonance (SPR), and circular dichroism (CD) were employed to characterize LB-A–GLP-1R interactions. The involvement of the GLP-1R/cAMP/PKA pathway and downstream mediators was examined using cellular assays, gene knockout, and Western blotting, with emphasis on FOXO1. LB-A exhibited the strongest binding affinity for GLP-1R among the analogs and significantly reduced blood glucose levels in mice. It formed hydrogen bonds and hydrophobic interactions with key residues (e.g., LYS197) and induced conformational changes in GLP-1R. LB-A activation of GLP-1R upregulated cAMP, PKA, and pCREB, suppressed PTEN/FOXO1 signaling, and subsequently stimulated insulin secretion. LB-A represents a novel small molecule agonist that ameliorates hyperglycemia in diabetic mice through specific activation of the GLP-1R/cAMP/PKA/pCREB/PTEN/FOXO1 pathway.

## 1. Introduction

Diabetes is a systemic metabolic disorder that has exhibited a continuous rise in global prevalence over recent decades, emerging as a major public health concern. According to the latest data from the International Diabetes Federation (2025), approximately 589 million adults aged 20–79 years (11.1% of the population) were living with diabetes as of 2024, with projections estimating that this will increase to 853 million (13%) by 2050 [1]. Glucagon-like peptide-1 receptor (GLP-1R) agonists, such as the peptide-based drug semaglutide, have emerged as cornerstone therapies in the management of diabetes. However, despite their potent hypoglycemic effects, peptide GLP-1R agonists encounter significant clinical limitations. The high incidence of gastrointestinal adverse events and requirement for non-parenteral administration contribute significantly to poor long-term patient compliance. To address these shortcomings associated with peptide agonists, researchers are increasingly focusing on small molecules in the hope of discovering novel therapeutic options. Nevertheless, numerous challenges remain unresolved with small-molecule GLP-1R agonists. Firstly, there exists a notable efficacy gap when compared to peptide agonists. This discrepancy may be attributed to the simpler structure of small molecules, which often results in lower affinity than that observed with peptide drugs and less robust and stable binding interactions with GLP-1R. In addition, small-molecule drugs exhibit relatively low bioavailability, which poses challenges in maintaining their concentrations within the effective therapeutic range in the body. Additionally, potential adverse reactions present another challenge that warrants careful consideration; small molecules may exhibit a higher propensity for interaction with non-target molecules, thereby increasing the risk of unintended side effects [2,3,4]. Despite these aforementioned deficiencies, small-molecule agonists represent a promising avenue for addressing existing limitations associated with GLP-1R therapies. Consequently, research into this class of compounds remains critically important and urgent.

Natural products, renowned for their unique structural diversity and broad biological activities, have historically served as an important resource for drug discovery. Several natural compounds, including chalcones, flavonoids, and polyphenols, have been reported to mimic key hydrogen-bond interactions of GLP-1, offering potential advantages in GLP-1R binding. Notably, rosmarinic acid and resveratrol have been shown to alleviate hyperglycemia in diabetic mouse models, partly through activation of GLP-1R-mediated signaling pathways [5,6]. Loureirin B (LB), a chalcone-derived natural product, has been identified by our group as a potential GLP-1R activator that promotes insulin secretion in Ins-1 pancreatic β-cells [7]. However, the presence of multiple methoxy groups in LB’s structure leads to strong hydrophobic interactions, which constrain its adaptability within the GLP-1R binding pocket. Traditional strategies for optimizing natural products typically focus on improving individual physicochemical properties, often without considering receptor conformational dynamics in a systematic manner.

In this study, we selected agglutinin B as the lead compound and rationally modified its structure by introducing hydroxyl and carboxyl groups to enhance hydrogen-bonding capacity and aqueous solubility, while preserving its chalcone backbone to maintain receptor compatibility and increase affinity with the receptor, aiming to achieve superior efficacy and pharmacokinetic parameters. We evaluated the bioactivity of the modified compound as a small-molecule GLP-1R activator through a series of in vitro and in vivo assays. Additionally, we performed preliminary mechanistic investigations to elucidate its primary molecular targets and regulatory pathways. Our findings offer novel insights into the design of small-molecule GLP-1R agonists and provide a promising lead for the development of improved therapeutic agents for diabetes management.

## 2. Results

### 2.1. Screening and Synthesis of the Molecular Structures of Loureirin B Analogs

#### 2.1.1. Screening of the Optimal Molecular Structure

**Figure 1:**
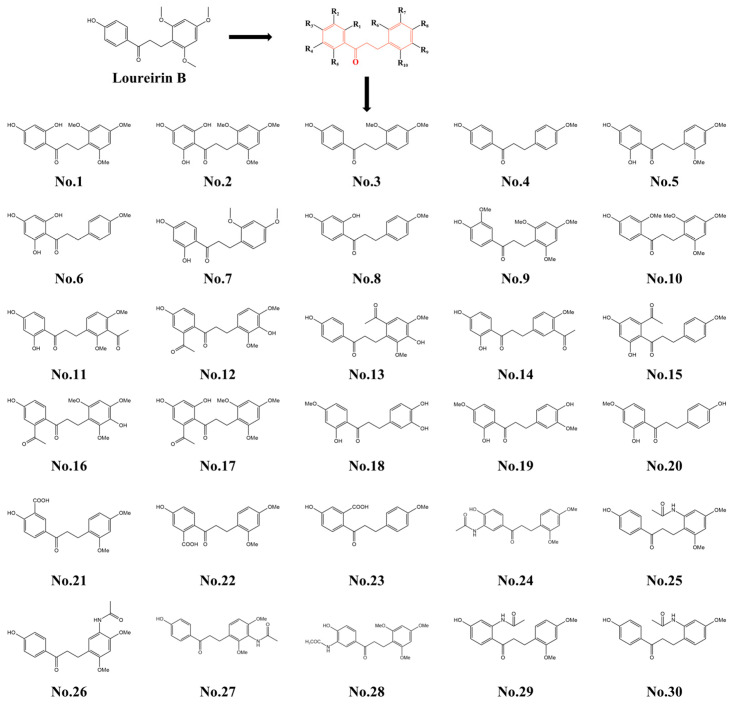
LB analogs structures.

Using Loureirin B as the leading compound, a series of thirty derivatives (Figure 1) were designed based on structural modification strategies aimed at optimizing their interactions with targets related to diabetes treatment. The structural transformations were primarily guided by the following design principles: (1) retain the chalcone core framework; (2) introduce carboxyl or hydroxyl groups to enhance the water solubility of the molecules while promoting hydrogen bond formation with the target; (3) Incorporate proton able amino groups to improve target binding affinity and increase water solubility via protonation; (4) eliminate the methoxy group to reduce steric hindrance, thereby enhancing molecular binding affinity to the target and simultaneously decreasing hydrophobicity for improved solubility. Using this systematic approach to structural optimization, a total of 30 novel Loureirin B derivatives were designed and synthesized (Figure 1 and Table S1).

Molecular docking analyses were conducted to evaluate the binding affinity of 30 LB-A analog structures with GLP-1R, and the results are presented in Table 1. From the data in this table, it is evident that compound No. 7 exhibited the highest score (−cDocker energy = 59.87), surpassing LB, suggesting that compound No. 7 may possess a stronger interaction with GLP-1R. Consequently, we designated compound No. 7 LB-A for further experimental validation.

#### 2.1.2. The Interaction Between LB-A and GLP-1R

**Figure 2:**
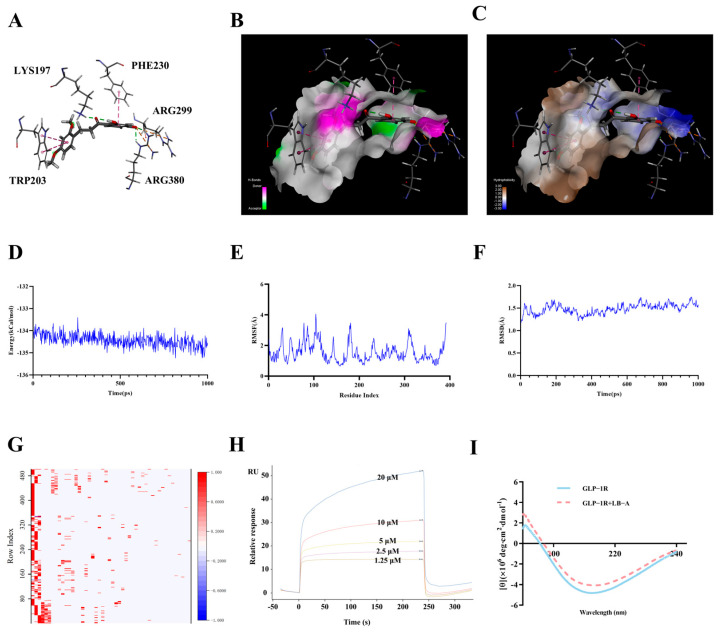
Simulation of the interaction between LB-A and GLP-1R. (**A**) Interaction between LB-A and GLP-1R (3D). (**B**) Hydrogen bonding. (**C**) Hydrophobic effect. (**D**) Binding energy. (**E**) RMSF. (**F**) RMSD. (**G**) Hydrogen bond heat map. (**H**) SPR of LB-A with GLP-1R. (**I**) CD of LB-A with GLP-1R.

The docking results of LB-A with GLP-1R were further analyzed, and the findings are presented in Figure 2. As illustrated in Figure 2A, the key amino acid residues involved in the interaction between GLP-1R and LB-A include lysine (LYS197), tryptophan (TRP203), phenylalanine (PHE230), and arginine (ARG299, ARG380). Further analysis indicated that the primary interactions between GLP-1R and LB-A were mediated by hydrogen bonds (Figure 2B) and hydrophobic interactions (Figure 2C), with significant hydrogen bond lengths measuring at 1.70 Å, 1.74 Å, and 2.04 Å, respectively. To further assess the interaction level between these two entities, a molecular dynamics simulation of the complex formed by LB-A and GLP-1R was conducted over a duration of 1000 ps. It is noteworthy that the total energy associated with this complex also demonstrated considerable stability throughout. The energy remains consistently within a range from −135.23 kcal/mol to −133.14 kcal/mol during intervals spanning from t = 0 ps to t = 1000 ps, with no substantial differences noted among various conformations examined. It was observed that the root mean square fluctuation (RMSF) for this complex remained relatively stable throughout; each conformation exhibited an RMSF ranging from approximately 0.65 Å to as high as about 4.05 Å without any significant deviations occurring—indicating that amino acid residues within this complex maintained relative stability during simulations (Figure 2E). During the initial phase of this simulation—specifically within the first 300 ps—the root mean square deviation (RMSD) fluctuated between values of approximately 1.17 Å to 1.68 Å before gradually stabilizing around an average RMSD value of approximately 1.57 Å; ultimately leading to stabilization over time with a total average RMSD recorded at about 1.48 Å (Figure 2F). Additionally, hydrogen-bond heat map analysis (Figure 2G) revealed relatively strong hydrogen-bond interactions across different conformations. Collectively, these results suggest that LB-A forms a highly stable complex with GLP-1R, thereby indicating its potential as a target for therapeutic intervention involving LB-A. Consequently, following these analyses, LB-A was selected for chemical synthesis (Figure S1); post-synthesis verification confirmed structural conformity, as depicted in Supplementary Figure S2, leading to its designation as LB-A for subsequent experimental validation.

To investigate the impact of LB-A on the structure of GLP-1R and to elucidate their interaction, Surface Plasmon Resonance (SPR) and Circular Dichroism (CD) analyses were performed. Figure 2H illustrates the SPR findings regarding the effect of varying concentrations of LB-A on GLP-1R’s structure. The data indicate that as the concentration of LB-A increases, there is a linear rise in Response Unit (RU) values, reaching 52 at a concentration of 2 × 10^−5^ mol/L. The binding constant derived from SPR measurements was determined to be 4.87 × 10^4^ L/mol, suggesting an interaction between LB-A and GLP-1R. Figure 2I reveals a broad peak within the circular dichroism spectrum of GLP-1R in the range of 210–220 nm, indicating that β-folding constitutes a significant component of its secondary structure. Following the addition of LB-A, both ellipticity and spectral intensity at the peak around 222 nm exhibited marked decreases, signifying a loss of α-helical content during their interaction and subsequent alterations to GLP-1R’s secondary structure. Specific changes are detailed in Table 2. Post-interaction with LB-A resulted in decreased α-helical content for GLP-1R, while increasing proportions were observed for β-folding, β-turns, and random coil structures. This suggests that LB-A can modify the secondary structure of GLP-1R, potentially due to their interactive relationship. And when compared to LB, it was observed that the druggability of LB-A was superior, and its efficacy in promoting insulin secretion was notably enhanced (Figure S3 and Table S2).

### 2.2. The Impact of LB-A on Diabetic Mice

**Figure 3:**
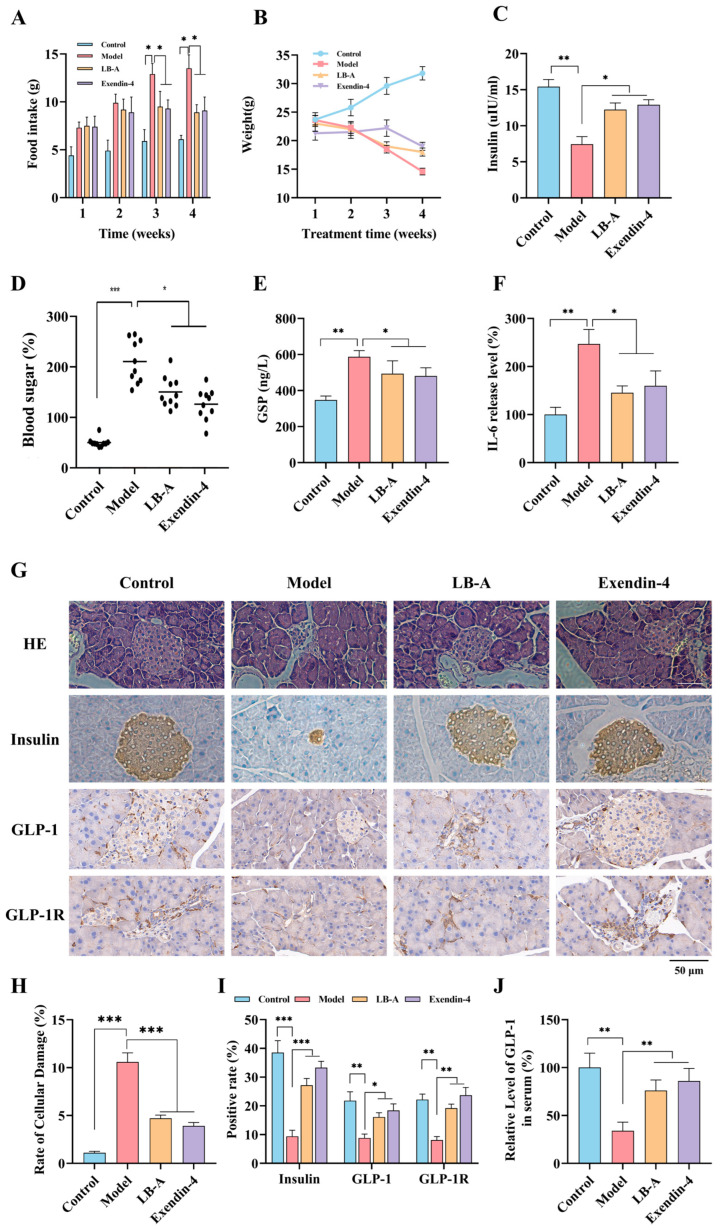
The impact of LB-A on diabetic mice. (**A**) Food intake. (**B**) Weight. (**C**) Insulin secretion. (**D**) Blood sugar. (**E**) Glycated serum protein. (**F**) IL-6. (**G**) HE, Insulin, GLP-1R, and GLP-1 staining. (**H**,**I**) Quantification of HE, Insulin, GLP-1R, and GLP-1 staining. (**J**) GLP-1 level in serum. * *p* < 0.05, ** *p* < 0.01, *** *p* < 0.001.

To further assess the in vivo therapeutic effects of LB-A, diabetic mice were administered LB-A, and changes in various indicators, including food intake and body weight, were monitored. The results are presented in Figure 3. As illustrated in Figure 3A,B, the food intake of mice in the Model group was significantly higher than that of healthy mice in the control group during weeks 3–4. Additionally, the body weight of mice in the Model group was lower compared to that of their healthy counterparts. Notably, food intake in mice treated with LB-A was significantly reduced relative to that observed in the Model group and approached levels seen in healthy mice. However, despite this reduction, the body weight of mice receiving LB-A treatment remained lower than that of those in the control group. Furthermore, insulin secretion was markedly decreased in mice from the Model group when compared to those from the control group; however, treatment with LB-A effectively restored insulin levels to within normal ranges (Figure 3C).

On the other hand, the blood glucose levels in the Model group of mice were significantly higher than those in the healthy group. Following four weeks of LB-A treatment, a notable decrease in blood glucose levels was recorded among diabetic mice. Concurrently, LB-A also reduces glycosylated serum protein (GSP) levels in these diabetic mice (Figure 3D,E). As illustrated in Figure 3F, there was a significant increase in interleukin-6 (IL-6) levels following the onset of diabetes, indicating a relatively severe inflammatory response. However, after administration of LB-A, IL-6 levels decreased markedly, suggesting an alleviation of inflammation (Figure 3F). Histological examination through hematoxylin and eosin (HE) staining of pancreatic tissue from mice revealed substantial tissue damage within the pancreas of those in the Model group. This included structural loss (as indicated in Figure 3G–I) and cellular necrosis. Although islet damage did not fully revert to a healthy state post-LB-A treatment, there was a significant improvement. Insulin staining results further demonstrated that insulin secretion was considerably diminished in diabetic mice; however, LB-A treatment effectively restored insulin levels. Glucagon-like peptide-1 (GLP-1) and its receptor GLP-1R are critical mediators involved in insulin secretion processes. Staining and subsequent observation showed reduced expression levels for both GLP-1 and GLP-1R in pancreatic tissues from the Model group mice; conversely, their expression significantly increased following LB-A treatment. This finding aligns with observed changes regarding insulin secretion dynamics. Furthermore, our findings indicate that the serum levels of GLP-1 in mice were significantly elevated following LB-A treatment (Figure 3J). Collectively, these results indicate that LB-A has potential therapeutic effects by enhancing insulin levels and alleviating symptoms associated with diabetes mellitus in affected murine models. Moreover, we have also identified that LB-A exhibits outstanding pharmacokinetic properties (Figure S3 and Table S3).

### 2.3. The Impact of LB-A on Ins-1 Cells

**Figure 4:**
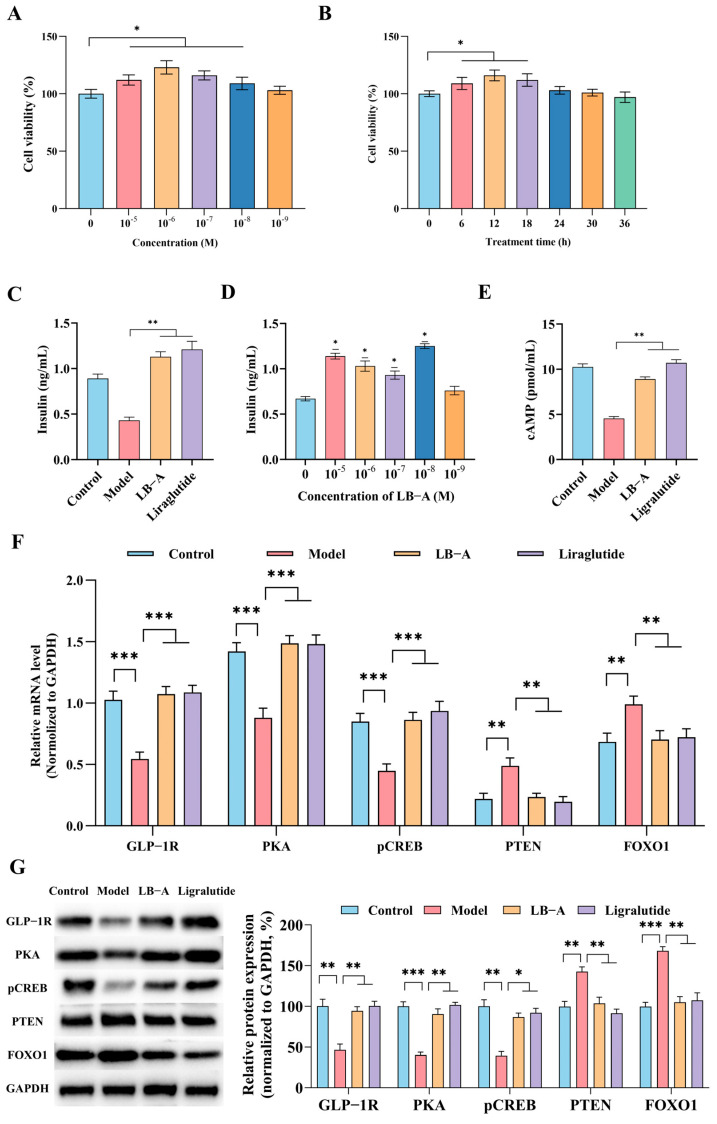
The impact of LB-A on Ins-1 cells. (**A**) Cell viability (at different concentrations of LB-A). (**B**) Cell viability (at different treatment times). (**C**) The level of insulin. (**D**) The level of insulin (different concentrations of LB-A). (**E**) The level of cAMP. (**F**) The expression of GLP-1R-related genes. (**G**) The expression of GLP-1R-related proteins. * *p* < 0.05, ** *p* < 0.01, *** *p* < 0.001.

To investigate the effects of LB-A on insulin secretion via GLP-1R, ins-1 cells were utilized as a model to analyze the regulatory impact of LB-A on the downstream signaling pathways associated with GLP-1R (Figure 4). Initially, ins-1 cells were incubated with varying concentrations and treatment durations of LB-A. It was observed that LB-A at concentrations ranging from 10^−5^ to 10^−9^ mol/L exhibited no significant cytotoxicity towards Ins-1 cells. Furthermore, within the concentration range of 10^−5^ to 10^−8^ mol/L, it could also stimulate cell growth to some extent (Figure 4A). Conversely, when the concentration of LB-A was set at 10^−8^ mol/L, there was no evident toxicity observed in Ins-1 cells even after continuous co-incubation for a duration of 36 h (Figure 4B). Subsequent analysis of insulin secretion revealed that LB-A significantly enhanced insulin secretion compared to the control group, and demonstrated an effect similar to that of LB. Specifically, when administered at concentrations between 10^−5^ to 10^−8^ mol/L, LB-A effectively stimulated insulin secretion in Ins-1 cells (Figure 4C,D). cAMP serves as a crucial intracellular second messenger for GLP-1R activation. As illustrated in Figure 4E, cAMP levels in the Model group were markedly reduced; however, following treatment with LB-A, cAMP content in diabetic cells showed a significant increase—indicative of GLP-1R activation. The results obtained from qPCR and Western blot analyses (Figure 4F,G) directly indicated that expression levels of GLP-1R in the Model group cells were significantly diminished. Treatment with LB-A ameliorated this condition by restoring GLP-1R expression levels back into normal ranges. Additionally, expressions of PKA and pCREB within Model group cells decreased substantially; conversely, both markers exhibited significant increases in expression levels within the LB-A treated groups. The trends of PTEN and FOXO1 were the opposite. In the Model group, the expression levels of PTEN and FOXO1 in the cells increased significantly. However, following treatment with LB-A, these levels decreased markedly compared to the Model group and approached those of the control group, remaining at a relatively low level. These findings suggest that LB-A does not exert toxic side effects on Ins-1 cells and can enhance insulin secretion in diabetic cells. This insulin-promoting effect may be mediated through the activation of GLP-1R.

### 2.4. LB-A Enhances Insulin Secretion Through the Activation of GLP-1 Receptor

**Figure 5:**
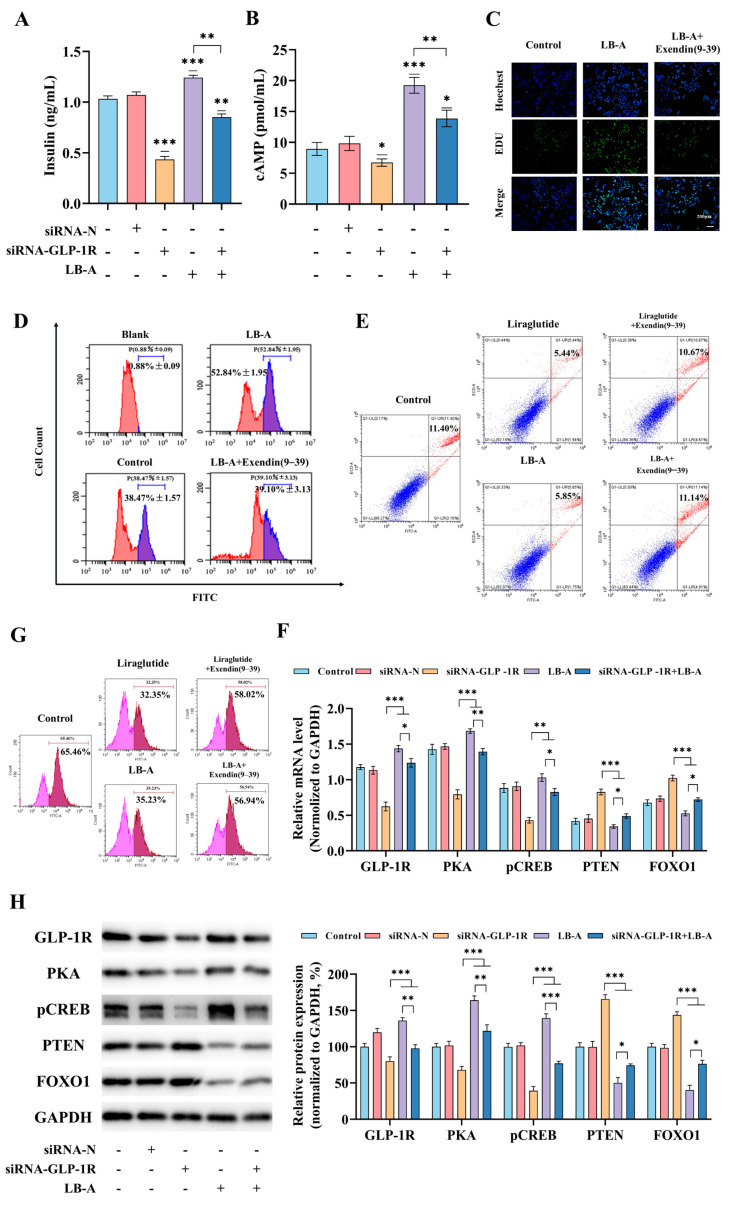
The impact of LB-A on cellular activity following the inhibition of GLP-1R. (**A**) The level of insulin. (**B**) The level of cAMP. (**C**,**D**) Cell proliferation. (**E**) Apoptosis. (**F**) ROS. (**G**) Modifications at the gene level. (**H**) Alterations in protein expression. * *p* < 0.05, ** *p* < 0.01, *** *p* < 0.001.

To further elucidate the role of GLP-1R in the insulin-secretion-promoting effects of LB-A, we evaluated the impact of LB-A on Ins-1 cells while inhibiting GLP-1R activity (Figure 5). As shown in Figure 5A, LB-A significantly enhances insulin levels. However, this stimulatory effect is diminished upon inhibition of GLP-1R. A similar trend was observed for cAMP levels. Treatment with LB-A alone resulted in an increase in cAMP content, but following GLP-1R inhibition, the enhancing effect of LB-A on cAMP was also partially attenuated (Figure 5B). Further analysis revealed that LB-A promotes the proliferation of Ins-1 cells; nevertheless, when GLP-1R was inhibited, there was a notable decrease in cell proliferation rates (Figure 5C,D). Conversely, LB-A significantly reduced apoptosis in Ins-1 cells. However, after inhibiting GLP-1R, there was a marked increase in apoptosis in these cells, accompanied by a corresponding reduction in the protective effects of LB-A (Figure 5E). Additionally, treatment with LB-A led to decreased ROS levels within Ins-1 cells; this effect was substantially weakened following GLP-1R inhibition (Figure 5F). Results from qPCR and Western blot analyses (Figure 5G,H) demonstrated that LB-A markedly increased expression levels of *GLP-1R*, *PKA*, and *pCREB* while concurrently reducing expressions of PTEN and FOXO1. This response was significantly diminished upon inhibition of GLP-1R—resulting in decreased expressions of GLP-1R, PKA, and pCREB alongside significant increases in PTEN and FOXO1 expressions. Collectively, these findings indicate that the insulin-stimulating effects exerted by LB-A are mediated through activation of GLP-1R.

### 2.5. The Role of FOXO1 in the Promotion of Insulin Secretion by LB-A

**Figure 6:**
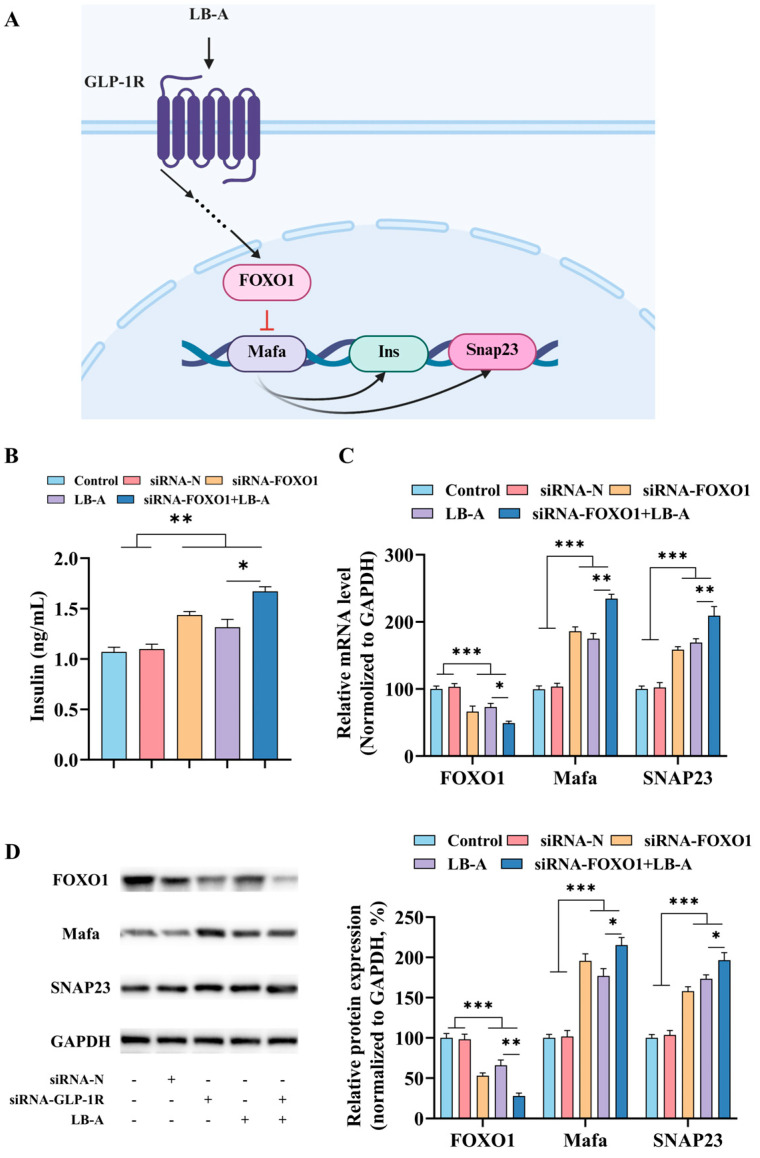
The impact of LB-A on cellular activity following the inhibition of FOXO1. (**A**) The potential functions of FOXO1. (**B**) The level of insulin. (**C**) Modifications at the gene level. (**D**) Alterations in protein expression. * *p* < 0.05, ** *p* < 0.01, *** *p* < 0.001.

Furthermore, the synthesis and secretion of insulin are contingent upon the activation of genes by transcription factors. In previous experiments, FOXO1 was identified as a significant transcription factor whose expression levels were altered in response to LB-A treatment. This finding suggests that FOXO1 may play a crucial role in mediating the effects of LB-A. To investigate the specific function of FOXO1 in the insulin-promoting secretion process induced by LB-A, this study inhibited its gene expression and subsequently observed the effects of LB-A. The results are presented in Figure 6. As shown in Figure 6A, following FOXO1 inhibition, there was an increase in insulin levels within Ins-1 cells. Moreover, upon addition of LB-A, it was noted that insulin content further escalated. Quantitative PCR (qPCR) analysis (Figure 6B) and Western blot (WB) (Figure 6C) were conducted on FOXO1, along with related genes Mafa and SNAP23, to assess changes in their gene and protein expressions. The data indicate that LB-A effectively inhibits *FOXO1* gene expression while simultaneously promoting *Mafa* and *SNAP23* expression; notably, this enhancing effect is amplified after inhibiting FOXO1. These findings suggest that LB-A can elevate protein levels of Mafa and SNAP23 through downregulation of FOXO1 expression, ultimately contributing to enhanced insulin secretion.

## 3. Discussion

### 3.1. The Molecular Mechanism Underlying the Interaction Between the Structural Optimization of LB-A and GLP-1R

The pharmacological treatment of diabetes has consistently been a prominent topic in scientific research globally, yielding numerous significant advancements. Nevertheless, existing antidiabetic medications do not fully address the clinical needs of patients. Natural products have historically served as a valuable source for developing therapeutics against various major diseases, including diabetes. Studies indicate that many natural small molecules exhibit hypoglycemic activity. For instance, rosmarinic acid has been shown to enhance glucose homeostasis in diabetic mice by promoting fatty acid β-oxidation and simultaneously reducing cholesterol and triglyceride levels in both plasma and liver [8]. Additionally, the polyphenolic small molecule Resveratrol can restore insulin sensitivity in skeletal muscle cells, augment glucose uptake, reverse insulin resistance, and improve GLUT4 translocation [9]. Flavonoid compounds such as Baicalin and Dihydromyricetin demonstrate beneficial effects on glucose tolerance, enhance insulin sensitivity, and lower blood glucose levels in diabetic mice [10,11]. The mechanism underlying these natural products involves extensive hydrogen bond interactions formed between hydroxyl groups within their structures and amino acids present in GLP-1R. This interaction stabilizes the active conformation of the receptor while triggering downstream signal transduction pathways; thus, suggesting that hydroxyl groups play a crucial role. Loureirin B is identified as a flavonoid compound characterized by its relatively stable chalcone structure [12]. Furthermore, it contains multiple methoxy groups which have been reported to inhibit cell proliferation [13]. Therefore, in this study, we designed a series of compounds based on the structure of LB. By retaining its chalcone core and modifying the number and position of methoxy groups while introducing other electron-donating or -withdrawing groups such as hydroxyl groups, we aimed to enhance their pharmacological properties. Among these compounds, compound No. 7 (LB-A) exhibited the strongest interaction with the target GLP-1 receptor (GLP-1R). In comparison to LB, the structure of LB-A incorporates an additional hydroxyl group while simultaneously reducing one methoxy group, which increases the polarity of the molecule. This modification is expected to strengthen hydrogen bond interactions with the protein target, thereby improving its pharmacological efficacy. The ADMET results further indicated that LB-A demonstrated superior solubility, likely attributable to the introduction of hydroxyl groups. These newly added hydroxyl groups enhanced hydrogen bonding between LB-A and water molecules, resulting in increased solubility. Moreover, pharmacokinetic studies revealed that LB-A was absorbed relatively quickly within biological systems; it achieved higher concentrations in a shorter time frame while exhibiting a slow elimination rate. This characteristic allows for prolonged presence in circulation.

GLP-1R is a prototypical G protein-coupled receptor. Upon activation, its structural conformation undergoes changes that generate signals and activate downstream signaling pathways. When GLP-1 binds to GLP-1R, the N-terminal of GLP-1 is anchored within the transmembrane domain (TMD) pocket, while the central region of GLP-1 is encircled by extracellular loops ECL1, ECL2, and TM2. This interaction relies on a polar network established by LYS197. Additionally, residues THR298, ARG299, and TYR205 further stabilize the binding of GLP-1 to GLP-1R [14]. Moreover, during G protein coupling processes, intracellular loop 2 (ICL2) interacts with the GαN helix; PHE376 and ARG380 are crucial for this interaction [15]. Previous studies have demonstrated that LB binds to a pocket located in the extracellular domain (ECD) of GLP-1R primarily through interactions with amino acids such as ARG34, GLY35, ARG121, and LYS113. This binding mode bears similarities to those observed for Boc5 and WB4-24 [7,16]. LB-A exhibits strong hydrogen bonding interactions along with hydrophobic forces involving key amino acids (LYS197, TRP203, PHE230, ARG299, and ARG380) within GLP-1R while engaging deeper into both ECD and TMD pockets. The binding characteristics resemble those of full agonists RGT1383 and PF-06882961 as well as partial agonist LY3502970. Such binding enhances cAMP generation significantly [17,18,19]. The differences in binding affinity between LB-A and LB may be attributed to an increase in hydroxyl groups coupled with a decrease in methoxy groups. The former enhances molecular polarity as well as electron-donating capacity upon interaction with GLP-1R; conversely, the latter reduces both molecular weight and volume, facilitating access into deeper pockets. Results from RMSD and RMSF analyses confirmed that the complex structure formed between LB-A/GLP-1R remains relatively stable. This stable binding mode, which transcends the initial molecule docking and has been validated through long-term dynamic simulations, suggests that LB-A may exhibit a prolonged duration of action and a more stable therapeutic effect in vivo. This stability could mitigate the uncertainty regarding drug efficacy that arises from conformational fluctuations of the receptor. Furthermore, this evidence supports the notion that LB-A may serve as an excellent potential GLP-1R agonist. Furthermore, surface plasmon resonance alongside circular dichroism assays indicated that LB-A can induce alterations in the secondary structure of GLP-1R—providing additional evidence for their interaction. The conformational reprogramming of GLP-1R induced by LB-A may be crucial for the activation of the receptor’s downstream signaling pathways. Differences to the conformational changes elicited by the natural ligand GLP-1, LB-A, as a small molecule, appear to stabilize a specific active conformation of GLP-1R. This stabilization selectively activates signaling pathways that promote insulin secretion (such as cAMP/PKA) while potentially mitigating side effects associated with other pathways. This characteristic of “biased excitability” may be pivotal in enhancing the efficacy of GLP-1R and could represent a unique advantage of LB-A, thereby offering valuable insights for future research directions.

### 3.2. Pharmacodynamic Assessment of LB-A

GLP-1 receptor agonists have long been established as commonly utilized hypoglycemic agents in clinical practice. However, the majority of currently available related drugs are peptide-based, such as Exenatide and Liraglutide, which necessitate self-injection by patients, leading to suboptimal patient compliance. Although oral Semaglutide was approved for marketing in 2019 and has demonstrated significant reductions in HbA1c levels and body weight among individuals with type 2 diabetes mellitus (T2DM), it is associated with a higher incidence of gastrointestinal adverse reactions, including nausea, vomiting, and diarrhea [20]. In light of these limitations, the development of non-peptide GLP-1 receptor small molecule agonists has emerged as a promising therapeutic avenue. Boc5 represents the first non-peptide GLP-1 receptor agonist [21], capable of enhancing insulin secretion in isolated rat pancreatic islet tissue while exhibiting notable hypoglycemic effects in db/db mice. Nevertheless, its lower oral bioavailability has hindered its overall efficacy. WB4-24, a novel analog of Boc5, retains similar hypoglycemic properties but still falls short regarding optimal oral bioavailability [22]. In this study, we report the screening and synthesis of a novel small-molecule GLP-1 receptor agonist designated LB-A. This compound exhibited significant pharmacological activity and effectively ameliorated pathological damage to pancreatic islet tissue in diabetic mice; this improvement was primarily characterized by the restoration of structural integrity within pancreatic islets. Furthermore, LB-A facilitated increased insulin secretion while reducing fasting blood glucose levels in diabetic mice to near-normal ranges. Meanwhile, LB-A has also demonstrated the potential to alleviate chronic inflammation associated with diabetes and to upregulate GLP-1R in mice. After activation, GLP-1R not only performs functions such as stimulating glucose-dependent insulin secretion and inhibiting glucagon release to regulate blood glucose levels, but also exerts effects that promote β-cell proliferation, inhibit apoptosis, and enhance pancreatic islet cell function [23]. Consequently, the amelioration of pancreatic islet tissue injury in mice may represent a potential efficacy of LB-A. Simultaneously, chronic low-grade inflammation is recognized as one of the significant characteristics of diabetes. It is closely associated with insulin resistance and dysfunction of islet cells. Inflammatory factors such as IL-6 can impair the functionality of islet cells, thereby accelerating the progression of diabetes [24]. The anti-inflammatory properties of LB-A may also contribute to improving β-cell function from an alternative perspective. The restoration of GLP-1 may be attributed to the remission of diabetes within the mice themselves. These findings suggest that LB-A could exert specific reparative and protective effects on damaged pancreatic islet cells by enhancing the functionality of the GLP-1/GLP-1R axis, which may play a crucial role in the long-term management of diabetes. Notably, unlike conventional GLP-1 receptor agonists used clinically (such as liraglutide), treatment with LB-A did not significantly impact body weight among diabetic mice. This distinctive pharmacodynamic characteristic may be attributed to the selective activation of the signaling pathway, which is influenced by the specificity of its molecular structure. However, the precise mechanism requires further elucidation through subsequent experiments.

Compared to LB, the druggability of LB-A has been enhanced, and it exhibits a superior hypoglycemic effect. However, regarding the body weight of mice, LB-A did not demonstrate significant improvement. This may be attributed to the distinct G protein coupling preferences of LB-A and its weak activation of downstream pathways associated with weight reduction. This suggests that there is still potential for further optimization in the structure of LB-A. New compounds could be synthesized by coupling LB-A with other small molecules (such as curcumin), which may enhance their efficacy and contribute positively to weight management in mice. Additionally, such couplings might improve the interaction between LB-A and other cAMP-mediated targets, thereby further augmenting its hypoglycemic effects. On another note, the half-life of LB-A is 5.21 h. While this duration meets basic requirements, a longer-lasting effect would be desirable in diabetes treatment. Furthermore, an extended half-life could facilitate continuous receptor activation, prolong feelings of satiety, and enhance weight loss outcomes. Therefore, when pursuing further structural modifications, it may be essential to focus on extending the half-life of new molecules or developing sustained-release formulations (for instance, through nanocarrier systems).

### 3.3. The Regulatory Mechanism of LB-A

The regulatory mechanism of GLP-1R is complex and multifaceted. Upon activation, GLP-1R triggers a significant increase in the intracellular second messenger cAMP, which subsequently activates protein kinase A (PKA). This cascade ultimately promotes insulin synthesis and secretion while inhibiting glucagon release [25]. Additionally, GLP-1R can mediate GLUT4 signal transduction by influencing Ca^2+^ transport, leading to the activation of the PI3K/AKT signaling pathway. This process enhances insulin secretion and ameliorates insulin resistance in the liver and muscle tissues [26]. Que et al. discovered that GLP-1R activation may also result in down-regulation of CREB signals alongside up-regulation of inflammation-related proteins. In this context, Liraglutide administration could reverse these changes by re-establishing CREB signaling and reducing associated inflammatory responses, thereby achieving therapeutic effects for osteoarthritis in rat models [27]. Furthermore, studies have indicated a correlation between GLP-1R and PTEN; however, the specific regulatory mechanisms involved remain unclear [28,29]. Similar to the classical GLP-1R signal transduction pathway, LB-A significantly elevates intracellular cAMP levels through selective activation of GLP-1R. This mechanism may represent a crucial molecular basis for LB-A’s pharmacological effects. The accumulation of cAMP further stimulates the PKA signaling pathway, resulting in a marked increase in phosphorylation levels of the transcription factor CREB. The activation of CREB may facilitate insulin synthesis-related signaling pathways by down-regulating PTEN expression. Ultimately, this series of molecular events leads to a substantial enhancement in insulin secretion levels and achieves an impressive hypoglycemic effect.

As previously mentioned, LB-A enhances the levels of cAMP, a widely recognized messenger molecule that not only mediates GLP-1R signaling but is also implicated in various other signaling pathways. DPP4 has been shown to inhibit the transduction of GLP-1/GLP-1R signals. Research indicates that DPP4 can induce angiogenic damage in rats, resulting in diastolic left ventricular dysfunction, a process associated with decreased cAMP levels. Inhibition of DPP4 reverses this damage and alleviates the condition of the rats [30]. GCG analogs significantly prolong the duration of cAMP signal transduction; this effect may stem from GIPR, GCGR, and GLP-1R interactions [31]. Following LB-A activation of GLP-1R, there is a marked increase in cAMP content within pancreatic islet cells. From a multi-target perspective, the elevation in cAMP levels may not solely result from GLP-1R activation but could also involve other diabetes-related therapeutic targets such as DPP4, GIPR, and GCGR. This indicates that LB-A may interact with additional targets beyond GLP-1R. Our further research has demonstrated that LB-A also engages with Cxcr2, leading to downstream signal transduction and ultimately reducing oxidative stress [32]. This multi-target effect presents a double-edged sword. On one hand, LB-A may alleviate diabetes symptoms through the synergistic action of multiple related targets. On the other hand, activating more targets increases the potential for adverse reactions. This observation prompts new considerations for subsequent investigations into LB-A, specifically, how to confine its action to primary therapeutic targets relevant to diabetes and its complications while minimizing interaction with other targets. Addressing this challenge will be crucial for future research endeavors.

In addition to its action on cAMP, the activation of GLP-1R may also lead to the recruitment of β-arrestin [33]. Studies have indicated that certain GLP-1R agonists, upon activating GLP-1R, primarily result in a significant accumulation of cAMP with comparatively less recruitment of β-arrestin. This biased signal transduction could contribute to the prolonged retention of GLP-1R on the plasma membrane, thereby facilitating sustained effects [34,35]. However, it remains unclear whether this biased signal transduction might induce other complications. LB-A can enhance cAMP accumulation via GLP-1R activation, which is beneficial for lowering blood glucose levels. Nonetheless, it is still uncertain whether LB-A will affect β-arrestin levels or cause biased excitation of GLP-1R. Further experimental investigations are necessary to clarify these aspects in future research.

Although extensive research has been conducted on GLP-1R, its complex mechanism of action remains incompletely understood, particularly regarding its influence on nuclear pathways and its direct regulation of insulin synthesis and secretion at the DNA level. Xu et al. [36] demonstrated that exenatide could restore the systemic Th17/Treg balance in db/db mice during diabetes progression by modulating the FOXO1 pathway. This intervention reduced Th17 cell infiltration into pancreatic islet tissue, thereby alleviating islet inflammation. Similarly, Cai et al., in their investigation of liraglutide’s effects on diabetic cardiomyopathy, identified a correlation between FOXO1 expression and the GLP-1/GLP-1R axis. They reported that liraglutide significantly reduced the expression of both FOXO1 and MURF1, effectively reversing the progression of heart failure [37]. Furthermore, semaglutide treatment has been shown to increase GLP-1R and acetate levels in the hypothalamus. Elevated hypothalamic acetate activates GPR43, which subsequently inhibits POMC neuron activation via FOXO1, ultimately contributing to reduced food intake in obese mice [38]. In our study, we observed that LB-A activation of GLP-1R led to a significant reduction in the cellular protein expression levels of the transcription factor FOXO1. To verify the critical role of FOXO1 in the mechanism of LB-A action, we employed siRNA-mediated FOXO1 knockdown. The results revealed that the insulin-secretion-stimulating effect of LB-A was markedly attenuated (*p* < 0.05), underscoring the pivotal role of FOXO1 in the LB-A signaling pathway. Further investigation demonstrated that downregulation of FOXO1 resulted in a significant upregulation of Mafa expression. As a key transcriptional regulator of insulin synthesis and secretion, Mafa can directly activate the transcription of insulin-related genes by binding to the insulin gene promoter region. Additionally, it facilitates the fusion of insulin-secreting vesicles with the cell membrane by enhancing Snap23 expression. These findings suggest that LB-A may exert synergistic effects on pancreatic β-cell function, enhancing both insulin synthesis and secretion.

### 3.4. Limitations

Although this study has yielded promising results, several issues remain that warrant improvement and further validation in future research. Firstly, while a preliminary comparison was conducted between LB-A and both LB and Exendin-4, the analysis lacks sufficient comparisons with existing marketed GLP-1R agonists (such as semaglutide) and small molecule agonists currently in clinical research stages (such as orforglipron). And there is a lack of more “head-to-head” comparison data. This is of great help for clarifying the efficacy of LB-A. Secondly, this study primarily focused on the interaction between LB-A and GLP-1R but did not investigate whether LB-A exerts activating or inhibitory effects on other related GPCRs (such as glucagon receptors GCGR, GIP receptors, etc.). Given that members of the GLP-1R family share certain structural similarities, it is plausible that LB-A may also interact with analogous receptors. Such off-target effects could potentially lead to adverse reactions. Additionally, the disease models employed in this study were limited to a diabetic mouse model and an Ins-1 cell model. Neither of these models fully replicates human diabetes symptoms; thus, they can only serve as preliminary experimental validations. Future studies should incorporate larger animal models for more robust verification. Despite these limitations, the findings regarding LB-A offer valuable insights into the development of GLP-1R small molecule agonists.

## 4. Materials and Methods

### 4.1. Materials and Reagents

The 6-week-old male C57BL/6 mice were obtained from GemPharmatech Co., Ltd. (Jiangsu, China). The high-fat diet, comprising 60 kcal% fat calories (Ingredients: lard, 31.7%; casein, 25.8%; maltodextrin, 16.2%; sucrose, 8.9%; etc.), was procured from Dyets Biotechnology (Wuxi) Co., Ltd. (Wuxi, China). Streptozotocin (STZ) was sourced from Aladdin Reagent (Shanghai) Co., Ltd. (Shanghai, China). Ins-1 cells were acquired from Shanghai Zhong Qiao Xin Zhou Biotechnology Co., Ltd. (Shanghai, China). Furthermore, MEM medium, fetal bovine serum (FBS), and penicillin-streptomycin were obtained from Cytiva Biotechnology (Hangzhou) Co., Ltd. (Hangzhou, China). The remaining conventional reagents were all acquired from Sinopharm Chemical Reagent Co., Ltd. (Shanghai, China).

### 4.2. Methods

#### 4.2.1. Construction and Screening of Loureirin B Derivatives

The depiction of chemical structures is accomplished utilizing ChemDraw (version 20.0.0.41) software. Molecular docking, molecular dynamics simulations, and ADMET predictions were conducted utilizing Discovery Studio 4.1 software.

Molecular docking: The structure of the GLP-1R (PDB ID: 7S15) protein was obtained from the Protein Data Bank (PDB). This structure served as the receptor, while each derivative functioned as a ligand. The cDocker module within DS was employed to simulate molecular docking between the receptor and each derivative.

Molecular dynamics simulation: First, obtain the receptor-ligand complex from the molecular docking step. Subsequently, perform molecular dynamics simulations utilizing the simulation section in DS. Finally, analyze the results to derive key features such as RMSD and RMSF.

#### 4.2.2. Synthesis of Loureirin B Analog (LB-A)

3 g 1-(2,4-bis(benzyloxy)phenyl)ethan-1-one and 1.6 g of 2,4-Dimethoxybenzaldehyde, 50 mL of methanol was added to dissolve in a single-necked vial, and 1.5 g of potassium hydroxide was added while stirring, and it was placed in an oil bath to raise the temperature to 60 °C, the reaction was carried out overnight, detected by TLC, and the reaction was completed, cooled down to room temperature, filtered, and a yellow solid intermediate product (SM) was obtained. Add 3.2 g SM and 320 mg 10% palladium and 4.2 g ammonium formate in a single-necked vial, add 20 mL anhydrous methanol, stir, raise the temperature to 60 °C, react for 2 h, TLC detection, the raw material disappeared, filtration, the filtrate spin-dried, and the white solid was obtained by column chromatography (PE–EA = 2:1).

#### 4.2.3. Construction of Diabetic Model Mice

After one week of adaptive feeding, 6-week-old male C57BL/6 mice were divided into a control group and a high-fat and high-sugar group. The control group was fed a conventional diet, while the high-fat and high-sugar group received a diet rich in fats and sugars. Four weeks later, the model group mice were intraperitoneally injected with streptozotocin (STZ) at a dosage of 45 mg/kg, dissolved in 450 mM phosphate buffer. In contrast, the control group was administered an equivalent volume of phosphate buffer via intraperitoneal injection for one week. Fasting blood glucose levels were monitored throughout this period; an FBG level ≥ 11.0 mmol/L was deemed indicative of successful diabetic mouse model establishment. The animal study protocol was approved by the Ethics Committee of Shanghai University (ECSHU 2022-063, date: 2 March 2022).

The successfully modeled mice were then randomly assigned to three groups: the Model group, the LB-A treatment group, and the positive control group. All groups continued to receive a high-fat and high-sugar diet for four additional weeks. The LB-A treatment group received daily intraperitoneal injections of LB-A at a dose of 30 mg/kg in phosphate buffer. Meanwhile, the positive control group was administered Exendin-4 at the same dosage using identical methods, whereas the Model group continued receiving only phosphate buffer injections over this four-week period. The control group’s dietary regimen remained normal throughout.

During this experimental phase, various parameters, including fasting blood glucose levels, body weight, food intake, and other relevant indicators, were continuously monitored in all groups. At the conclusion of these observations, all mice underwent anesthesia prior to the collection of blood samples and organs such as the pancreas for subsequent analyses.

#### 4.2.4. Detection of Physiological Indicators in Mice

The concentrations of insulin, GSP, and IL-6 in the blood of mice were measured using specific detection kits for each component: insulin detection kits (E-EL-M1382, Elabscience Biotechnology Co, Ltd. Wuhan, China), GSP detection kits (ml037458, Shanghai Enzyme-linked Biotechnology Co., Ltd. Shanghai, China), and IL-6 detection kits (PI326, Beyotime Biotech, Shanghai, China).

#### 4.2.5. Tissue Staining in Mice

HE staining: Paraffin sections were dewaxed to remove the paraffin, followed by hydration with water. The sections were then stained with hematoxylin and subsequently treated with a bluing solution. After washing with water, the samples were dehydrated and stained using an eosin staining solution. Following dehydration, the sections were sealed for preservation. Microscopic observations of the images were conducted and recorded accordingly.

Insulin (Servicebio GB12335-100, 1:1000), GLP-1 (Servicebio GB123493-100, 1:1000), and GLP-1R (Servicebio GB113881-100, 1:1000) staining: The paraffin-embedded sections were deparaffinized and rehydrated, followed by antigen retrieval, serum blocking, incubation with primary antibody, subsequent incubation with secondary antibody, DAB chromogenic reaction, hematoxylin counterstaining for nuclear staining, mounting, and observation under a microscope.

#### 4.2.6. Pharmacokinetics

Rats (*n = 6*) were adaptively fed for one week. Prior to gavage, the rats were fasted for 12 h and allowed free access to water. Following intragastric administration of LB-A at a dose of 25 mg/kg (prepared in a 0.5% sodium carboxymethyl cellulose (CMC-Na) solution), blood samples of 0.2 mL were collected from the tail vein at the following time points: 0, 0.083, 0.17, 0.33, 0.5, 0.75, 1, 2, 4, 6, 8, 12, 18, and 24 h). The samples were placed in pre-heparinized centrifuge tubes and centrifuged at a speed of 5000 r/min for five minutes to obtain plasma samples. From each collection point, fifty μL of plasma was taken and mixed with five μL of internal standard solution. After pretreatment, detection and analysis were performed using an LC-MS instrument.

#### 4.2.7. SPR and CD

SPR: Use the GE Biacore T200 instrument in conjunction with the GESSA chip for this experiment. The chip was pre-treated by injecting 0.05 mol/L NaOH and 1 mol/L NaCl three times, with each injection lasting for 1 min. The flow rate during the coupling of LB-A was set at 5 μg/L/min. LB-A (1 × 10^−5^ mol/L) was diluted using the flow buffer prior to application. Samples were collected from the surface of the chip channel for analysis. The reaction temperature was maintained at 25 (±0.1 °C), while a flow rate of 30 μL/min was established throughout the procedure. To eliminate non-specific binding and account for volume effects induced by the buffer, measurements from the detection channel (LB-A) were subtracted from those obtained in the reference channel. PBS (0.05 mol/L, pH = 7.4) served as the running buffer. Various concentrations of LB-A were prepared to assess their binding affinity to GLP-1R through SPR experiments, which were conducted in triplicate to ensure reliability and reproducibility of results.

CD: Circular dichroism spectra of GLP-1R were recorded before and after the addition of LB-A at room temperature, covering the wavelength range of 190–230 nm. The concentration of GLP-1R used was 10^−5^ mol/L, prepared by diluting with a 10^−3^ mol/L mother liquor and water. LB-A was added to achieve a final concentration of 5 × 10^−7^ mol/L. The spectra were acquired with an instrumental scanning speed of 500 nm/min and a slit width of 2 nm. Each measurement was performed in triplicate. The obtained data were analyzed using DichroWeb to ascertain the changes in the secondary structure of GLP-1R protein induced by the interaction with LB-A [39].

#### 4.2.8. Ins-1 Cell Viability Assay

Ins-1 cells were starved with serum-free medium (inoculated at a density of 4 × 10^3^ cells/well density inoculated in 96-well plates) for 12 h, and then treated with different concentrations of LB-A for 24 h, 5 replicates for each treatment, repeated 3 times. Add 10 μL of CCK8 assay solution to each well, react for 45 min at 37 °C, measure OD450, and calculate cell viability according to the formula:

$$Cell\;viability\left(\%\right)=\frac{A\left(spiked\right)-A\left(blank\right)}{A\left(0spiked\right)-A\left(blank\right)}\times 100$$

where A (spiked) is the absorbance of LB-A-treated and added CCK8 reaction solution wells; A (0 spiked) is the absorbance of normal cell and CCK-8 reaction solution wells, and A (blank) is the absorbance of cell culture medium only wells.

#### 4.2.9. Detection of cAMP Level in Ins-1 Cells

After LB-A treatment of Ins-1 cells for 15 min, the cAMP-Glo™ kit was used to detect the changes in cAMP levels in Ins-1 cells.

#### 4.2.10. Ins-1 Cell Proliferation Assay

LB-A treated Ins-1 cells for 24 h, added 50 mM Edu (RiboBio, Guangzhou, China), and incubated for 3 h. After fixation and permeabilization, 100 μL 1 × Apollo^®^ reaction mixture was added and reacted for 30 min, and DAPI staining was performed. The cells were detected by fluorescence microscopy and flow cytometry.

#### 4.2.11. Ins-1 Cell Apoptosis Detection

LB-A treated Ins-1 cells for 12 h. Cells were collected, washed with PBS, and 100 μL of 1 × Binding Buffer to resuspend the cells; 5 μL Annexin V-Alexa Fluor 488 and 10 μL PI were added and mixed well. React for 10–15 min at room temperature, protected from light. 400 μL of 1 × Binding Buffer was added, mixed well, placed on ice, and the samples were detected by flow cytometry or fluorescence microscopy within 1 h. The samples were then analyzed by flow cytometry or fluorescence microscopy.

#### 4.2.12. Ins-1 Cell ROS Assay

ROS production was monitored by DCF fluorescence assay. Ins-1 cells were treated with LB-A (inoculated in 6-well plates at a density of 5 × 10^5^ cells/well) for 12 h. Serum-free medium with 10 μM DCFH-DA (2′,7′-dichlorofluorescent yellow bis-acetate) was added and incubated for 30 min, cells were collected and the fluorescence intensity was analyzed using flow cytometry.

#### 4.2.13. qPCR and WB

qPCR detection: Mice kidney RNA was extracted, and cDNA was obtained through reverse transcription. The resulting cDNA was subjected to qPCR analysis to determine the relative expression levels of GLP-1R, PKA, pCREB, PTEN, FOXO1, Mafa, and SNAP23 genes (Table 3).

Western blot: extraction and quantification of kidney tissue protein. The target protein was detected by Western blot, with electrophoresis conditions set at 400 mA for 65 min.

#### 4.2.14. Statistical Analysis

All the aforementioned experiments were conducted with five replicates. The data are expressed as the mean ± SD, and all assays for each independent treatment were performed in triplicate. Appropriate data were analyzed to determine statistical significance using one-way ANOVA (SPSS version 13.0, USA). Student’s t-test was used to examine differences between group means. * *p* < 0.05, ** *p* < 0.01, *** *p* < 0.001.

## 5. Conclusions

In this study, we designed and synthesized the small molecule LB-A through structural modification and screening of LB. Experimental results demonstrated that LB-A promotes insulin secretion in mice and significantly improves blood glucose levels as well as alleviates islet injury in diabetic mice. Molecular dynamics simulations, surface plasmon resonance, and circular dichroism analyses collectively confirmed a strong and stable interaction between LB-A and GLP-1R. Notably, the binding of LB-A induces changes in the secondary structure of GLP-1R. Further cell experiments validated the insulin-stimulating effect of LB-A, which operates by activating GLP-1R to regulate downstream signaling pathways involving cAMP/PKA/pCREB. Additionally, FOXO1 was found to play a crucial role in mediating the insulin-stimulating effects exerted by LB-A.

## Figures and Tables

**Table 1:** The simulation results of derivatives and GLP-1R, and the main active bonds.

No.	-cDocker Energy	Bone Length	Acting Force
1	56.54	2.06	Conventional Hydrogen Bond, Pi-Alkyl, Pi-Donor Hydrogen Bond
2	44.49	2.42	Carbon Hydrogen Bond, Conventional Hydrogen Bond, Pi-Cation
3	56.98	2.88	Conventional Hydrogen Bond, Pi-Pi T-shaped, Carbon Hydrogen Bond
4	58.22	2.06	Conventional Hydrogen Bond, Pi-Alkyl, Pi-Pi Stacked
5	56.37	2.09	Conventional Hydrogen Bond, Pi-Cation, Salt Bridge
6	55.69	2.20	Conventional Hydrogen Bond,
7	59.87	2.04	Conventional Hydrogen Bond, Pi-Pi Stacked, Pi-Cation
8	56.35	2.15	Conventional Hydrogen Bond, Carbon Hydrogen Bond
9	51.72	2.74	Carbon Hydrogen Bond, Pi-Pi Stacked, Pi-Alkyl
10	54.00	2.08	Conventional Hydrogen Bond, Pi-Cation
11	56.26	2.58	Carbon Hydrogen Bond, Conventional Hydrogen Bond, Pi-Alkyl
12	56.10	2.38	Carbon Hydrogen Bond, Pi-Pi Stacked
13	55.75	2.34	Carbon Hydrogen Bond, Conventional Hydrogen Bond
14	53.66	1.93	Conventional Hydrogen Bond, Pi-Pi Stacked,
15	52.10	1.82	Conventional Hydrogen Bond, Carbon Hydrogen Bond, Pi-Alkyl
16	52.30	2.18	Conventional Hydrogen Bond, Carbon Hydrogen Bond
17	55.54	2.25	Conventional Hydrogen Bond, Carbon Hydrogen Bond
18	30.97	2.42	Carbon Hydrogen Bond, Pi-Pi Stacked, Pi-Cation
19	20.91	2.2	Conventional Hydrogen Bond, Pi-Alkyl, Pi-Cation
20	28.19	1.94	Conventional Hydrogen Bond, Carbon Hydrogen Bond
21	51.67	1.71	Conventional Hydrogen Bond, Pi-Cation
22	53.05	1.95	Salt Bridge, Conventional Hydrogen Bond
23	54.49	1.89	Salt Bridge, Conventional Hydrogen Bond, Pi-Alkyl
24	55.64	2.09	Conventional Hydrogen Bond, Carbon Hydrogen Bond, Pi-Cation
25	49.13	1.86	Conventional Hydrogen Bond, Pi-Pi Stacked, Pi-Alkyl
26	52.25	2.06	Conventional Hydrogen Bond, Pi-Alkyl, Pi-Cation
27	53.98	2.07	Conventional Hydrogen Bond, Carbon Hydrogen Bond
28	52.51	2.19	Conventional Hydrogen Bond, Carbon Hydrogen Bond, Pi-Pi Stacked
29	33.78	2.14	Conventional Hydrogen Bond, Pi-Cation
30	48.92	2.28	Conventional Hydrogen Bond, Pi-Pi Stacked
LB [7]	40.42	2.33	Conventional Hydrogen Bond, Carbon Hydrogen Bond

**Table 2:** The impact of LB-A on the secondary structure of GLP-1R.

Structure	Regular α-Helix ^a^	Irregular α-Helix ^a^	Regular β-Folding ^a^	Irregular β-Folding ^a^	β-Turn ^a^	Random Coil ^a^
GLP-1R	12.2	10.8	24.2	10.5	16.6	25.7
GLP-1R + LB-A	11.4	10.6	25.6	9.9	17.1	26.3

^a^: the unit is (%).

**Table 3:** List of qPCR primers.

Genes	Forward (5′–3′)	Reverse (5′–3′)
*GPL-1R*	GGGCCAGTAGTGTGCTACAA	CTTCACACTCCGACAGGTCC
*PKA*	TACTTGGCCCCCGAGATTATC	GCGAAGAAGGGTGGGTAACC
*pCREB*	TGCCCCTGGAGTTGTTATGG	CTCTTGCTGCCTCCCTGTTC
*PTEN*	TGGATTCGACTTAGACTTGACCT	GGTGGGTTATGGTCTTCAAAAGG
*FOXO1*	CACCATGATGCAGCAGACGC	CAACTCCTTCAAGCCTCCAG
*Mafa*	CTTCAGCAAGGAGGAGGGTCATC	GCGTAGCCGCGGTTCTT
*SNAP23*	GCCACAGCATTTGTTGAGTTC	GCAGGAATCAAGACCATCACT
*GAPDH*	GGCAAGTTCAACGGCACAGT	TGGTGAAGACGCCAGTAGACTC

## Data Availability

The original contributions presented in this study are included in the article/Supplementary Materials. Further inquiries can be directed to the corresponding authors.

## Supplementary Materials

The following supporting information can be downloaded at: https://www.mdpi.com/article/10.3390/ijms262311548/s1.
