# Supplementary material for: From Phytochemistry to Metabolic Regulation: The Insulin-Promoting Effects of Loureirin B Analogous to an Agonist of GLP-1 Receptor
Source: Int J Mol Sci. 2025 Nov 28;26(23):11548. doi: 10.3390/ijms262311548 (PMC12692253; doi:10.3390/ijms262311548)
Supplement: Supplementary file 1 [file ijms-26-11548-s001.zip › ijms-3986848-supplementary.pdf]

# Supplementary Information

# From phytochemistry to metabolic regulation: the insulin-promoting effects of Loureirin B derivatives as novel allosteric modulators of GLP-1 receptor

**Haowen Fang[1, 5, #], Xiaodong Sun[2, #], Yanting Ding[3, 4, #], Siyuan Gu[5], Bing Niu[5, *], Qin Chen[5, *]**

1. School of Life Sciences, Shanghai University, Shanghai, P. R. China

2. Shanghai Engineering Research Center of Organ Repair, Joint International Research Laboratory of Biomaterials and Biotechnology in Organ Repair (Ministry of Education), School of Medicine, Shanghai University, Shanghai, P. R. China

3. Tongji Hospital, School of Medicine, Tongji University, Shanghai, P. R. China

4. Shanghai Biochip Co., Ltd., National Engineering Center for Biochip at Shanghai, Shanghai, PR China

5. School of environmental and chemical engineering, Shanghai University, Shanghai, P. R. China

# These authors contributed equally.

* Corresponding author at: School of Medicine, Shanghai University, Shanghai 200444, P. R. China. E-mail: sunxiaodong_cpu@163.com (Xiaodong Sun)

* Corresponding author at: School of Life Sciences, Shanghai University, Shanghai 200444, P. R. China. E-mail: bingniu@shu.edu.cn (Bing Niu)

* Corresponding author: School of Life Sciences, Shanghai University, Shanghai 200444, P. R. China. E-mail: chenqincc@shu.edu.cn (Qin Chen)

1. **Structural Design of LB Analogues**

| NO. | R1 | R2 | R3 | R4 | R5 | R6 | R7 | R8 | R9 | R10 | Formula |
|---|---|---|---|---|---|---|---|---|---|---|---|
| 1 | -OH | | -OH | | | $-OCH_3$ | | $-OCH_3$ | | $-OCH_3$ | $C_{18}H_{20}O_6$ |
| 2 | -OH | | -OH | | -OH | $-OCH_3$ | | $-OCH_3$ | | $-OCH_3$ | $C_{18}H_{20}O_7$ |
| 3 | | | -OH | | | $-OCH_3$ | | $-OCH_3$ | | | $C_{17}H_{18}O_4$ |
| 4 | | | -OH | | | | | $-OCH_3$ | | | $C_{16}H_{16}O_3$ |
| 5 | -OH | | | | -OH | | | $-OCH_3$ | | $-OCH_3$ | $C_{17}H_{18}O_5$ |
| 6 | -OH | | -OH | | -OH | | | $-OCH_3$ | | | $C_{16}H_{16}O_5$ |
| 7 | | | -OH | | -OH | $-OCH_3$ | | $-OCH_3$ | | | $C_{17}H_{18}O_5$ |
| 8 | -OH | | -OH | | | | | $-OCH_3$ | | | $C_{16}H_{16}O_4$ |
| 9 | | $-OCH_3$ | -OH | | | $-OCH_3$ | | $-OCH_3$ | | $-OCH_3$ | $C_{19}H_{22}O_6$ |
| 10 | $-OCH_3$ | | -OH | | | $-OCH_3$ | | $-OCH_3$ | | $-OCH_3$ | $C_{19}H_{22}O_6$ |
| 11 | | | -OH | | -OH | | | $-OCH_3$ | $-COCH_3$ | $-OCH_3$ | $C_{19}H_{20}O_6$ |
| 12 | | | -OH | | $-COCH_3$ | | | $-OCH_3$ | -OH | $-OCH_3$ | $C_{19}H_{20}O_6$ |
| 13 | | | -OH | | | $-COCH_3$ | | $-OCH_3$ | -OH | $-OCH_3$ | $C_{19}H_{20}O_6$ |
| 14 | | | -OH | | -OH | | | | $-OCH_3$ | $-COCH_3$ | $C_{18}H_{18}O_5$ |
| 15 | $-COCH_3$ | | -OH | | -OH | | | $-OCH_3$ | | | $C_{18}H_{18}O_5$ |
| 16 | | | -OH | | -COCH | $-OCH_3$ | | $-OCH_3$ | -OH | $-OCH_3$ | $C_{20}H_{22}O_7$ |
| 17 | -OH | | -OH | | -COCH | $-OCH_3$ | | $-OCH_3$ | | $-OCH_3$ | $C_{20}H_{22}O_7$ |
| 18 | | | $-OCH_3$ | | -OH | | | -OH | -OH | | $C_{16}H_{16}O_5$ |
| 19 | | | $-OCH_3$ | | -OH | | | -OH | $-OCH_3$ | | $C_{17}H_{18}O_5$ |
| 20 | | | $-OCH_3$ | | -OH | | | -OH | | | $C_{16}H_{16}O_4$ |
| 21 | | -COOH | -OH | | | | | $-OCH_3$ | | $-OCH_3$ | $C_{18}H_{18}O_6$ |

| | | | | | | | | | | |
|---|---|---|---|---|---|---|---|---|---|---|
| 22 | | -OH | | -COOH | | | $-OCH_3$ | | $-OCH_3$ | $C_{18}H_{18}O_6$ |
| 23 | -COOH | -OH | | | | | $-OCH_3$ | | | $C_{17}H_{16}O_5$ |
| 24 | | -OH | $-C_2NOH_4$ | | | | $-OCH_3$ | | $-OCH_3$ | $C_{19}H_{21}O_5N$ |
| 25 | | -OH | | | $-C_2NOH_4$ | | $-OCH_3$ | | $-OCH_3$ | $C_{19}H_{21}O_5N$ |
| 26 | | -OH | | | | $-C_2NOH_4$ | $-OCH_3$ | | $-OCH_3$ | $C_{19}H_{21}O_5N$ |
| 27 | | -OH | | | | | $-OCH_3$ | $-C_2NOH_4$ | $-OCH_3$ | $C_{19}H_{21}O_5N$ |
| 28 | | -OH | $-C_2NOH_4$ | | $-OCH_3$ | | $-OCH_3$ | | $-OCH_3$ | $C_{20}H_{23}O_6N$ |
| 29 | $-C_2NOH_4$ | -OH | | | | | $-OCH_3$ | | $-OCH_3$ | $C_{19}H_{21}O_5N$ |
| 30 | | -OH | | | $-C_2NOH_4$ | | $-OCH_3$ | | | $C_{18}H_{19}O_4N$ |

**Table S1**. LB analogues structures. The empty groups are all classified as -H.

## 2. Synthesis and Characterization of LB-A

LB-A was synthesized following the procedure outlined in supplementary **Figure S1A**. The structure of the synthesized compound was confirmed to align with expectations through mass spectrometry (**Figure S1B**), hydrogen spectroscopy (**Figure S1C**) and 13C NMR spectroscopy **(Figure S1D)**.

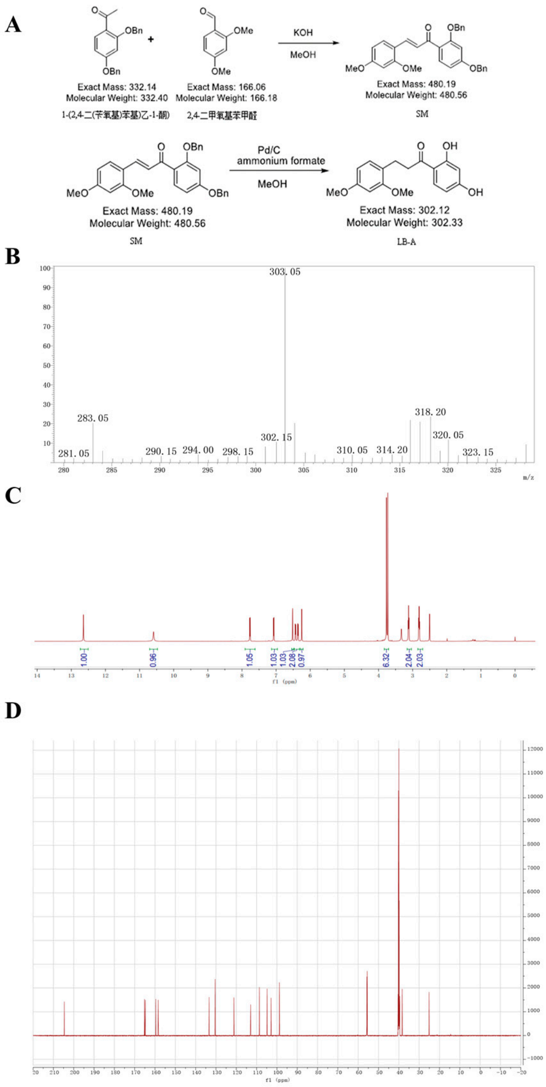


**Figure S1.** Synthesis and Characterization of LB-A. (A) Synthesis process. (B) The MS of LB-A. (C) The HNMR of LB-A. (D) The 13C NMR of LB-A.

**3. The druggability and insulin-stimulating effects of LB-A**

The druggability verification and preliminary assessment of the insulin-stimulating effect of LB-A were conducted (**Figure S2 and Table S1**). As illustrated in **Figure S2A**, both LB-A and LB fall within the confidence intervals for Absorping-95 and BBB95, while AlogP98 values are all below 4. This indicates that the predictions for both compounds are reliable (the results for 30 molecules regarding ADMET properties are provided in supplementary Figure 2). From the data shown in **Table S1**, it can be observed that SolubilityLB-A > SolubilityLB, BBBLB-A < BBBLB, CYP2D6LB-A > CYP2D6LB, and HepatotoxicLB-A > HepatotoxicLB. These findings suggest that LB-A exhibits relatively high solubility but has a slightly reduced ability to cross the blood-brain barrier. Consequently, this may mitigate its impact on the central nervous system while demonstrating minimal inhibitory effects on metabolism and lower hepatotoxicity. Furthermore, absorption levels for both compounds within the intestinal tract appear favorable. Additionally, plasma protein binding rates, lipid solubility levels, and polar surface area measurements remain within acceptable ranges. As depicted in **Figure S2B**, there is an enhancement in insulin-promoting secretion associated with LB-A compared to LB. Collectively, these results indicate that LB-A not only enhances drug efficacy relative to LB but also demonstrates improved drugability characteristics.

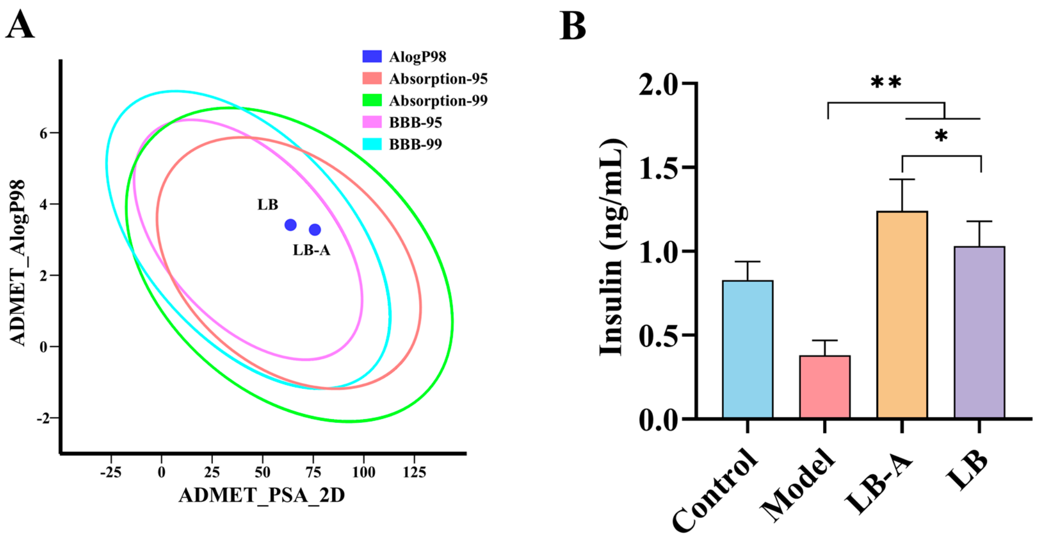


**Figure S2**. The druggability and insulin-stimulating effects of LB-A. (A)ADMET. (B)The level of insulin. *$p < 0.05$, $**p < 0.01$, $***p < 0.001$.*

**Table S2**. The Results of ADMET.

| | Solubility | BBB | CYP2D6 | Hepatotoxic | Absorption _Level | PPB | AlogP98 | PSA_2D |
|---|---|---|---|---|---|---|---|---|
| LB-A | -3.34 | -0.38 | 0.76 | 0.21 | 0 | 3.33 | 3.21 | 76.79 |
| LB | -3.81 | -0.12 | 1.25 | 0.51 | 0 | 4.25 | 3.43 | 64.91 |

**4. Pharmacokinetics of LB-A**

LB-A was administered to SD rats at a dose of 25 mg/kg. Blood samples were collected from the tail vein of the rats at following various time points: 0, 0.083, 0.17, 0.33, 0.5, 0.75, 1, 2, 4, 6, 8, 12, 18 and 24 h post-administration. The concentration of LB-A in rat plasma was analyzed using LC-MS techniques. The results are presented in **Table S2** and **Figure S3**. As illustrated in the figure, within the first half hour following administration, the plasma drug concentration rapidly increased from baseline to reach its peak (Cmax = 33.51 ng/mL), which corresponds with the peak time (Tmax = 0.5 hours) indicated in the table. Following oral administration of LB-A, it demonstrated rapid absorption in rats and efficiently completed its transit from the gastrointestinal tract into systemic circulation. In **Table S2**, the elimination half-life ($T_{0.5}$) is reported as being approximately 5.21 h; this aligns with observations noted during the "slow concentration attenuation phase" depicted in Figure 5 where plasma concentrations gradually declined from their peak value after administration—ultimately reaching 1.09 ng/mL by 24 h post-dose—indicating that LB-A is eliminated at a moderate rate within this animal model. The clearance rate was determined to be 0.11 mL/(g*h) which suggests relatively low clearance efficiency; this further corroborates that LB-A undergoes slow elimination processes within biological systems—a finding consistent with its pharmacokinetic profile characterized by an extended half-life. Overall, these findings indicate that following a single intragastric administration of 25 mg/kg LB-A, this compound exhibits pharmacokinetic properties marked by rapid absorption kinetics alongside significant total exposure while demonstrating moderate elimination rates and limited tissue distribution characteristics in SD rats.

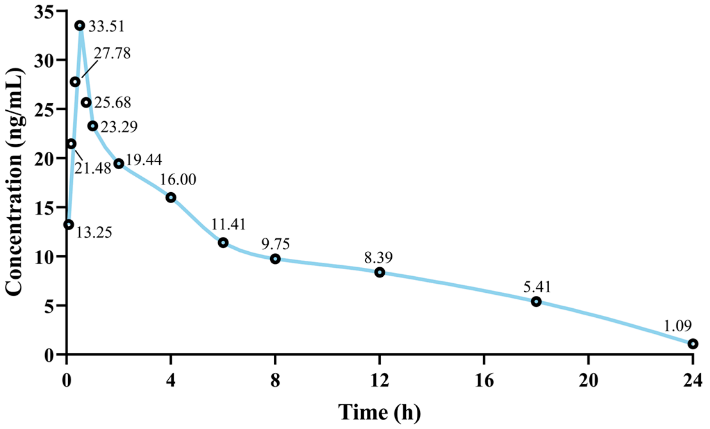


**Figure S3.** The pharmacokinetic curve of LB-A.

**Table S3**. The pharmacokinetic parameters of LB-A.

| No. | Parameters | Value |
|---|---|---|
| 1 | $AUC_{(0\text{-}t)}$/(ng/mL*h) | 227.22 |
| 2 | $AUC_{(0\text{-}\infty)}$/(ng/mL*h) | 235.47 |
| 3 | $C_{max}$/(ng/mL) | 33.51 |
| 4 | $T_{max}$/(h) | 0.5 |
| 5 | $T_{0.5}$/(h) | 5.21 |
| 6 | $V_d$/(mL/g) | 0.80 |
| 7 | Clearance rate /(mL/(g*h)) | 0.11 |
